# Investigation of Knowledge-Based Planning Models for Intensity-Modulated Proton Therapy With Limited Data for Brain Targets

**DOI:** 10.7759/cureus.81708

**Published:** 2025-04-04

**Authors:** Robert Kaderka, Yihang Xu, Gregory Azzam, Nesrin Dogan, Michael P Butkus

**Affiliations:** 1 Department of Radiation Oncology, University of Miami Miller School of Medicine, Miami, USA

**Keywords:** automated treatment planning, brain impt, brain tumour radiotherapy, knowledge-based planning, radiotherapy treatment planning

## Abstract

Background

Knowledge-based planning (KBP) in radiation therapy aims to improve the plan quality and planning efficiency. Limited training data complicate the development of KBP models for intensity-modulated proton therapy (IMPT). Clinicians face questions such as whether KBP validation is feasible on the training set and if small data sets substantially impact the resulting plan quality. This work describes the development of KBP models for brain IMPT while focusing on these key questions.

Materials and methods

A hundred and twenty brain IMPT plans were identified and 31 were selected for validation. Three KBP models were trained (a) KBP_V_ trained on the 31 validation plans; (b) KBP_O_, an open-loop model with 89 plans excluding the validation set; and (c) KBP_C_, a closed-loop model combining all the 120 plans. The 31 validation cases were re-planned using each KBP model. The resultant KBP plans were compared to manually-generated clinical plans and each other using dosimetric parameters and paired t-tests (p<0.05). Additionally, the manual and KBP_C _plans were evaluated through a blind physician review.

Results

Dosimetric differences between manual plans and the three KBP models were minimal. The KBP_V_, KBP_O_, and KBP_C_ models significantly reduced the clinical volume receiving 105% of the prescribed dose (V105%) to an average of 3.7%, 4.3%, and 4.5%, respectively compared to manual plans showing 15.2%. KBP_V_ reduced the mean dose to the left hippocampus (Hippocampus_L D_mean_) to 535±630 cGy compared to manual plans showing an average of 790±1077 cGy. The maximum dose to the pituitary gland (pituitary D_max_) for the manual plans was 1992±1951 cGy, which was reduced in both KBP_O_ and KBP_C_ to 1761±1832 cGy and 1801±1831 cGy, respectively. When the models were compared, KBP_O_ reduced the maximum dose to the optic chiasm (chiasm D_max_) to a greater extent than KBP_C _(2813 cGy vs. 2912 cGy, respectively).

In the blind physician review, all the KBP_C _plans were considered clinically acceptable. When stating a preference, the physician favored the KBP_C_ plans in 12 out of 31 cases. A tie was scored for 13 out of the 31 plans, while the manual plans were preferred in six out of the 31 cases.

Conclusion

All KBP models produced plans that were dosimetrically comparable to manually-generated reference plans. The blind physician review confirmed that the plans were clinically viable. Minimal dosimetric differences between the three different KBP models for IMPT suggest that KBP training and validation sets can be identical if needed. This finding supports the generation of KBP models for situations where the training data may be limited.

## Introduction

Knowledge-based planning (KBP) automates treatment planning in radiation therapy with a focus on increasing planning quality and efficiency while simultaneously reducing variability in the clinical workflow [1-5]. A database of previously treated plans is used to train a KBP model using machine learning. The model then predicts achievable dose-volume histograms (DVHs) for new plans and provides optimization objectives [6-11].

KBP for photon-based treatments has been extensively investigated and utilized in multiple clinics worldwide. In some clinics, KBP represents the starting point for the plan optimization process and has been demonstrated to be non-inferior to manual planning, while potentially decreasing overall planning time [12,13].

KBP has recently become commercially available for intensity-modulated proton therapy (IMPT), but its clinical use remains limited. Early adopters have focused on research applications of the technology in head-and-neck, prostate, gastroesophageal, and liver carcinoma [14-18] or for comparison between proton and photon treatment options [19].

One of the challenges of creating KBP models is the necessity of a large dataset for training and validation, which may not always be readily available in IMPT. Additionally, a concern in machine learning-based techniques is that using the training set for validation may lead to overfitting, reducing the model’s generalizability. These concerns exacerbate the issues of limited plan availability. Clinicians looking to implement KBP are therefore often faced with two key questions: Can the training set be used for model validation? And will the plan quality be compromised if the training model size is small?

This study describes the development of KBP models for IMPT of the brain and the base-of-skull. The models were trained on three different data sets with the aim of providing answers to the questions outlined above. A dosimetric evaluation was performed on the resulting plans. Additionally, a blind physician review was performed on the resulting KBP plans to assess whether they were acceptable for clinical treatment.

## Materials and methods

Creating the KBP models

The KBP models were built in RapidPlanPT (Eclipse ver. 16.1, Varian Medical Systems, Palo Alto, California). This commercial solution trains KBP models using a set of previously generated plans. A machine learning algorithm analyzes dose distributions and geometric parameters of the training set and creates estimations for achievable DVHs in the new plans. After the initial training, quality filtering is often suggested to improve the DVH predictions [7,10]. In the model, the user can set fixed optimization objectives that act as a template for every KBP-generated plan. Additionally, DVH predictions can be used to generate patient-specific optimization objectives. All optimization objectives can either receive an automatic priority or a priority set by the user at the model level. Previous studies have demonstrated that tuning the priorities on the optimization objectives can substantially impact the plan quality without any changes to the underlying training set [13].

Manual reference for IMPT planning

Our clinic employs robust optimization for IMPT treatment planning (Eclipse, ver 16.1, Varian Medical Systems, Palo Alto, California) to the clinical target volume (CTV) rather than planning target volume (PTV)-based optimization [20-22]. Planners generally add robust objectives to targets but may opt to add robustness on critical organs-at-risk (OARs). For targets in the brain and the base-of-skull, 3 mm setup uncertainties in all directions are coupled with proton range uncertainties of ±3.5% resulting in 12 uncertainty scenarios. After initial optimization, the planners may create localized optimization helper structures to locally increase or decrease the dose to cold or hot spots.

The planners are generally instructed to plan with a single-field optimization (SFO) with two to three coplanar fields. If the plan quality is not adequate, multi-field optimization (MFO) and/or non-coplanar fields may be utilized.

Patient cohort and the three KBP models

For this retrospective study, 120 patients were identified. A hundred and nine of them were treated with 180-200 cGy per fraction to a total dose of 5000-6000 cGy. These patients had a variety of diagnoses and the lesions were located within the brain or base-of-skull. The remaining 11 out of the 120 patients had recurrent glioblastomas treated to 3000-3500 cGy, with 300-350 cGy per fraction. The boost plans and brain plans for patients receiving craniospinal treatment were not included in these 120 plans.

The 120 plans were divided into groups to investigate the effects of validating on the training set and KBP model size. Out of the 120 plans, 31 were chosen for validation. The validation set was chosen randomly, but recurrent glioblastomas treated to 3000-3500 cGy were excluded from this set. Three KBP models were then trained based on this split. KBP_V _was a model trained on the 31 validation plans, this simulates a scenario where a clinic may have only limited data available for KBP training and testing. KBP_O_ was an open-loop model with the remaining 89 plans excluding the 31 validation cases. The use of open-loop models, where the training set is fully distinct from the validation set, is generally recommended for validating KBP routines as this approach eliminates bias of prediction to data already observed by the system and overfitting [11,23]. Finally, the KBP_C_ model represented a closed-loop model that included all the 120 patients, meaning that the training and validation sets had patients in common.

To isolate the effects of the training sets, optimization objectives were kept identical across the three models. These objectives are listed in Table 1 and were obtained by re-planning a subset of 20 plans using the KBP_O_ training set and iteratively adjusting the model objectives until the plan quality was deemed adequate. 

**Table 1:** Optimization objectives for knowledge-based planning (KBP) Optimization set for each of the KBP models in Eclipse (ver 16.1, Varian Medical Systems, Palo Alto, California). The robust column indicates which parameters were used for robust optimization. CTV: Clinical target volume; Rt: Right; Lt: Left

Parameter	Type	Vol	Dose	Priority	Robust
CTV high	Upper	0%	100%	0	
	Upper	0%	103%	180	Yes
	Lower	99.5%	99%	180	Yes
	Lower	99.9%	100%	200	
CTV intermediate	Upper	0%	100%	0	
	Upper	0%	103%	180	Yes
	Lower	99.9%	99%	180	
	Lower	99.0%	100%	200	Yes
Brain	Line (preferring target)	Generated	Generated	50	
Brain-CTV	Line (preferring target)	Generated	Generated	50	
Brainstem	Upper	0%	4800cGy	100	
	Upper	0%	5200cGy	180	
	Upper (fixed vol., generated dose)	0%	Generated	79	
	Line (preferring target)	Generated	Generated	50	
Chiasm	Upper	0%	4800cGy	100	
	Upper	0%	5200cGy	180	
	Upper (fixed vol., generated dose)	0%	Generated	79	
	Line (preferring target)	Generated	Generated	50	
Cochlea Lt	Mean		3000cGy	50	
	Mean		Generated	49	
	Line (preferring target)	Generated	Generated	50	
	Upper (fixed vol. generated dose)	0	Generated	49	
Cochlea Rt	Mean		3000cGy	50	
	Mean		Generated	49	
	Line (preferring target)	Generated	Generated	50	
	Upper (fixed vol. generated dose)	0	Generated	49	
Eye Lt	Upper	0%	4200cGy	75	
	Upper (fixed vol., generated dose)	0%	Generated	49	
	Line (preferring target)	Generated	Generated	50	
Eye Rt	Upper	0%	4200cGy	75	
	Upper (fixed vol., generated dose)	0%	Generated	49	
	Line (preferring target)	Generated	Generated	50	
Hippocampus Lt	Upper	0%	1200cGy	75	
	Upper (fixed vol., generated dose)	0%	Generated	99	
	Line (preferring target)	Generated	Generated	50	
Hippocampus Rt	Upper	0%	1200cGy	75	
	Upper (fixed vol., generated dose)	0%	Generated	99	
	Line (preferring target)	Generated	Generated	50	
Lacrimal gland Lt	Mean		1000cGy	50	
	Line (preferring target)	Generated	Generated	50	
Lacrimal gland Rt	Mean		1000cGy	50	
	Line (preferring target)	Generated	Generated	50	
Lens Lt	Upper	0%	500cGy	50	
	Upper (fixed vol., generated dose)	0%	Generated	49	
	Line (preferring target)	Generated	Generated	50	
Lens Rt	Upper	0%	500cGy	50	
	Upper (fixed vol., generated dose)	0%	Generated	49	
	Line (preferring target)	Generated	Generated	50	
Optic Nerve Lt	Upper	0%	5200cGy	200	
	Upper	0%	4700cGy	100	
	Upper (fixed vol., generated dose)	0%	Generated	99	
	Line (preferring target)	Generated	Generated	50	
Optic nerve Rt	Upper	0%	5200cGy	200	
	Upper	0%	4700cGy	100	
	Upper (fixed vol., generated dose)	0%	Generated	99	
	Line (preferring target)	Generated	Generated	50	
Pituitary	Upper	0%	1700cGy	50	
	Upper (fixed vol. generated dose)	0%	Generated	49	
	Line (preferring target)	Generated	Generated	50	
Ring	Line (preferring target)	Generated	Generated	50	
Skin	Line (preferring target)	Generated	Generated	100	
Spinal cord	Upper	0%	4100cGy	200	
	Upper (fixed vol., generated dose)	0%	Generated	99	
	Line (preferring target)	Generated	Generated	50	
Normal tissue objective	Manual			60	
	Distance from target border: 0.70 cm				
	Start dose 98%				
	End dose 30%				

Testing the KBP model and data analysis

The performance of the models was tested by re-planning the 31 validation plans using all the three models. The beam angles, choice of SFO/MFO, and robust optimization scenarios were kept identical to the manually-generated clinical plans. To emulate the tendency of the planners of reducing hotspots as a final step, the “Continue optimization” feature in the treatment planning system was used after initial KBP optimization with an additional 103% robust upper objective on the body at priority 500. The final plan was then normalized to match the same percentage of the volume of the target tissue receiving 100% of the prescribed dose (V100%) as the manual plan (typically, but not always, CTV V100%=95%).

The resulting KBP plans were compared to the manual reference plans and to each other. Comparisons included the standard DVH parameters evaluated for targets in the brain: maximum dose (D_max_) to the body, CTV, brainstem, eye, lacrimal glands, lens, optic chiasm, optic nerves, and pituitary gland. For cochlea and hippocampi, the mean dose (D_mean_) was analyzed. Additionally, CTV minimum dose (D_min_), CTV percentage of the volume of the target tissue receiving 105% of the prescribed dose (V105%), and robustness in terms of the worst-case CTV percentage of the planning target volume receiving at least 95% of the prescribed dose (V95%) for the 12 uncertainty scenarios were compared. Finally, the integral dose was evaluated in terms of volume of the body (in cubic centimeters or cc) that receives at least 100%, 50%, 30%, and 10% of the prescribed radiation dose, i.e. V100%, V50%, V30%, and V10%, respectively. The aggregate differences were tested for significance using paired t-tests (p<0.05).

Finally, the 31 plans resulting from the KBP_C_ model, along with corresponding manually-generated plans were presented to a physician for blind review. The physician was asked to assess whether the plans were clinically acceptable and to indicate which of the two plans was preferred, and was allowed to score a tie if there was no clear preference.

## Results

On our clinical server, initial optimization with KBP typically took around five minutes and continued optimization around two minutes. After the initial optimization, four out of 31 plans had a body maximum dose >5% compared to the manual plan. Using the “Continue optimization” feature with the upper objective on the body successfully reduced the hotspot in all 31 plans without negatively impacting other parameters. 

**Figure 1:**
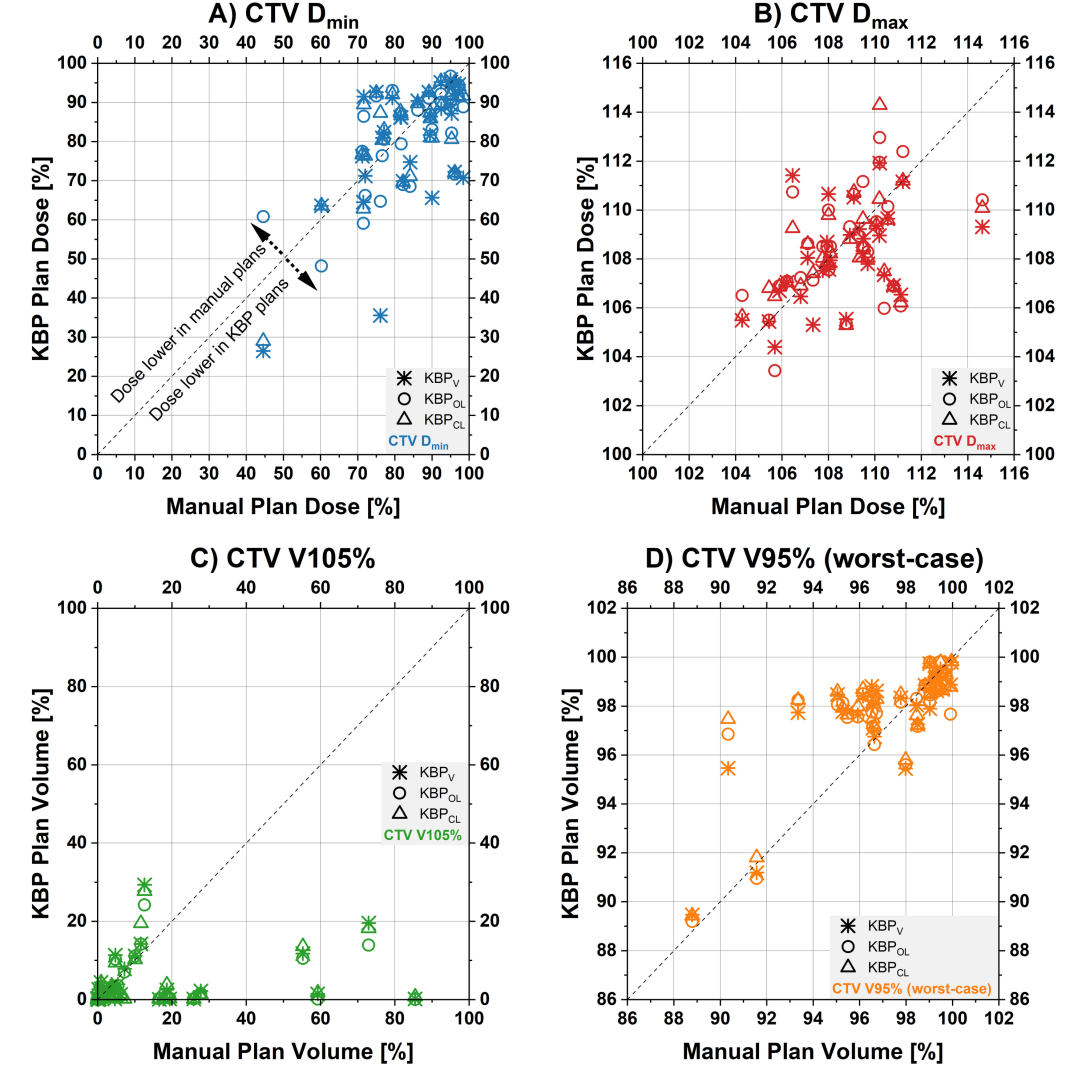
Dose-volume histogram (DVH) parameters for clinical target volume (CTV) Scatterplot of the CTV DVH parameters for the manual plans (values on x-axes) and the three different knowledge-based planning (KBP) models (values on y-axes, each model represented by its own symbol). Points on the diagonal line indicate DVH parameters for KBP plans were identical to the manual plans. Points below the equality line illustrate a lower dose by the KBP plan for this patient. A) CTV D_min_, B) CTV D_max_, C) CTV V105%, D) CTV V95% worst-case (measure of plan robustness) D_min_: minimum dose; D_max_: maximum dose; V105%: percentage of the volume of the target tissue receiving 105% of the prescribed dose; V95%: percentage of the planning target volume receiving at least 95% of the prescribed dose

**Figure 2:**
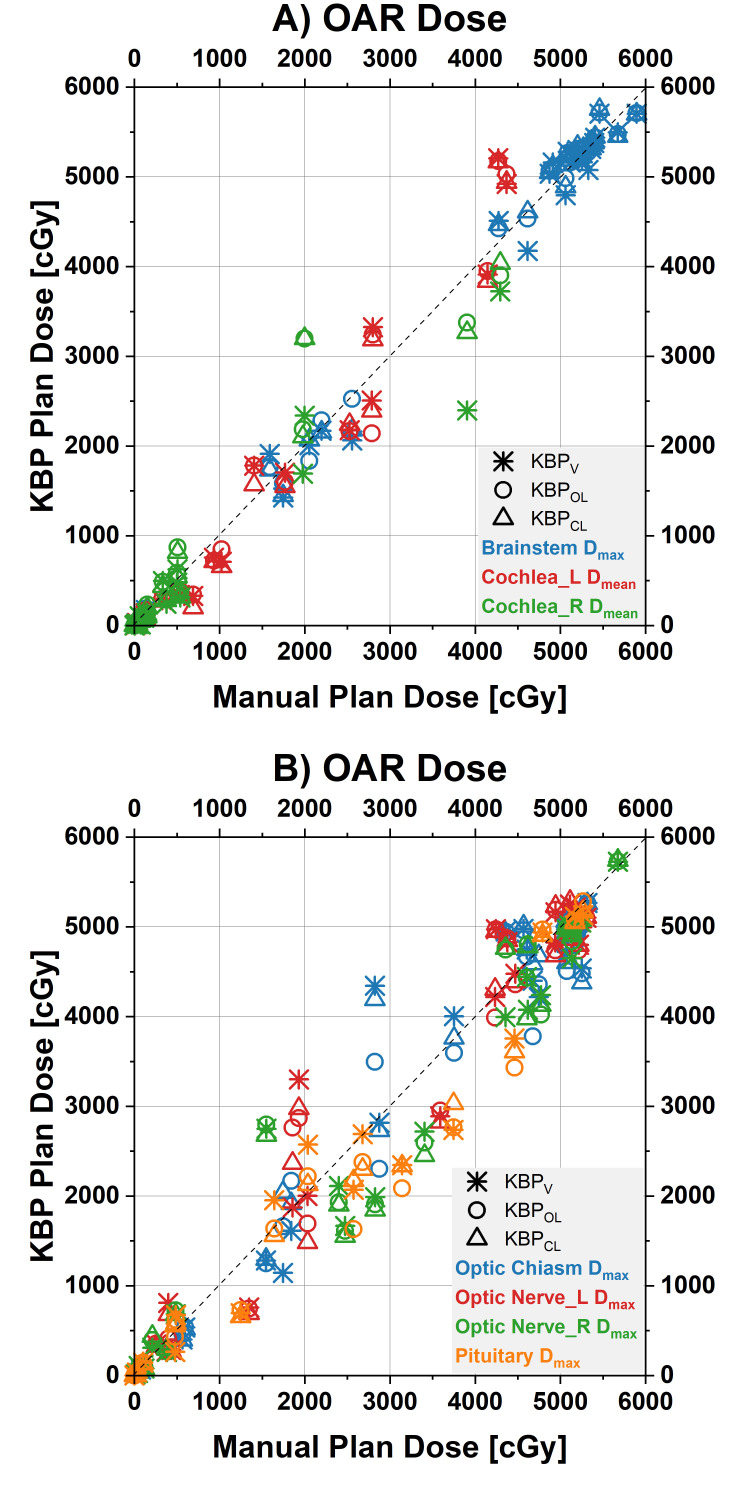
Dose-volume histogram (DVH) parameters for the brainstem and the optics Scatterplot of the clinical target volume (CTV) DVH parameters for the manual plans (values on x-axes) and the three different knowledge-based planning (KBP) models (values on y-axes, each model represented by its own symbol). Points on the diagonal line indicate DVH parameters for KBP plans were identical to the manual plans. Points below the equality line illustrate a lower dose by the KBP plan for this patient. A) D_max_ for the brainstem and D_mean_ for the cochlea, B) D_max_ for the optics and the pituitary D_max_: maximum dose; D_mean_: mean dose.

**Figure 3:**
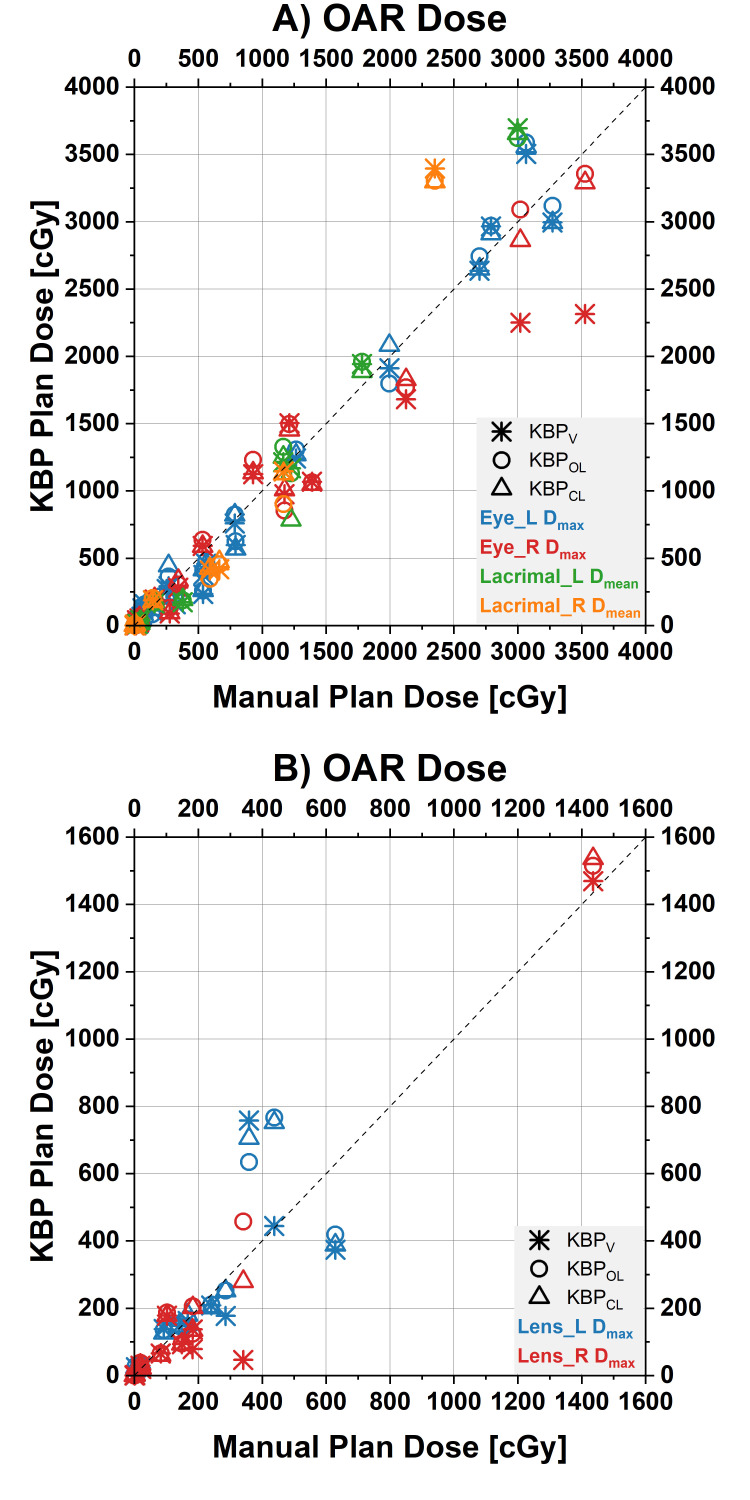
Dose-volume histogram (DVH) parameters for the eyes and the lenses Scatterplot of the clinical target volume (CTV) DVH parameters for manual plans (values on x-axes) and the three different knowledge-based planning (KBP) models (values on y-axes, each model represented by its own symbol). Points on the diagonal line indicate DVH parameters for KBP plans were identical to the manual plans. Points below the equality line illustrate a lower dose by the KBP plan for this patient. A) D_max _for the eyes and D_mean_ for the lacrimal glands, B) D_max_ for the lenses D_max_: maximum dose; D_mean_: mean dose.

**Figure 4:**
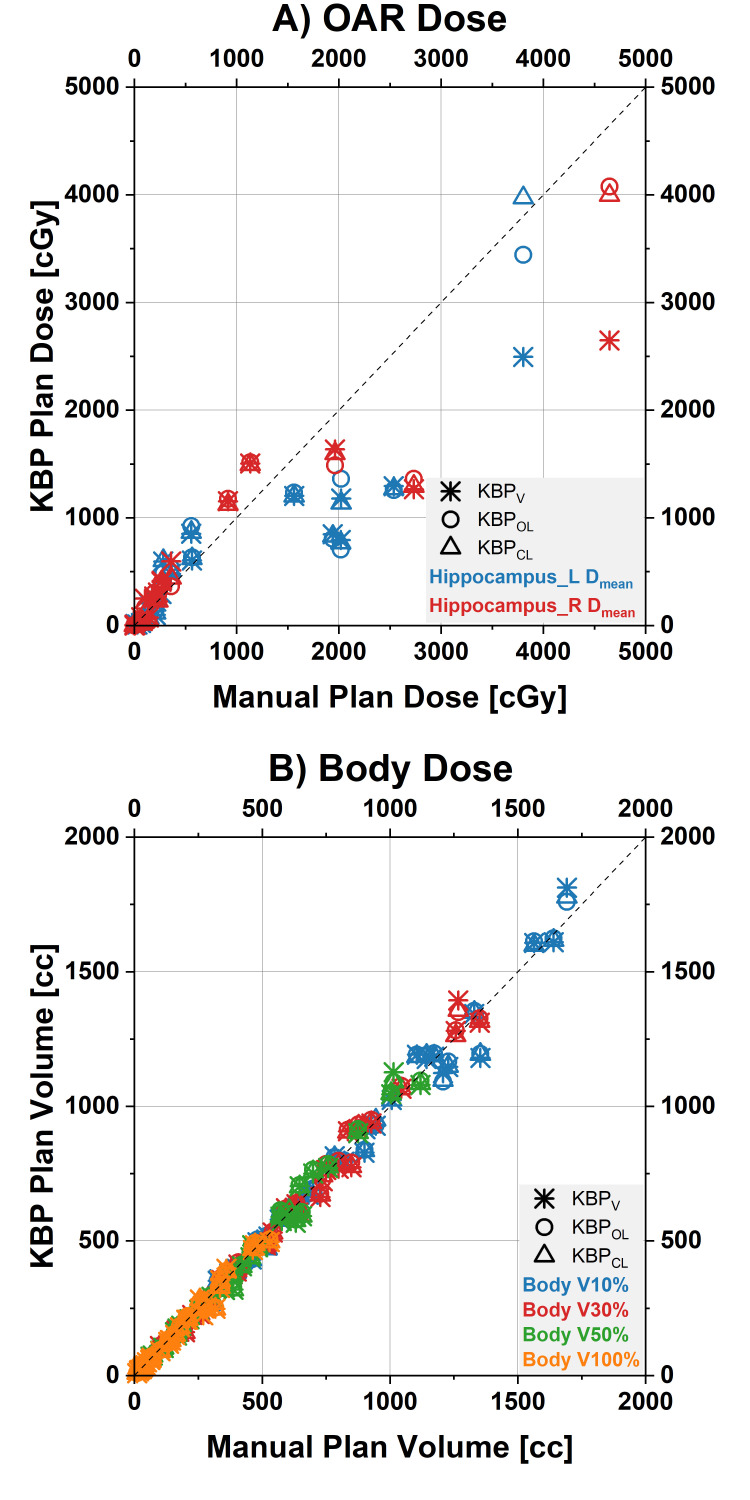
Dose-volume histogram (DVH) parameters for the hippocampus and the body Scatterplot of the clinical target volume (CTV) DVH parameters for the manual plans (values on x-axes) and the three different KBP models (values on y-axes, each model represented by its own symbol). Points on the diagonal line indicate DVH parameters for KBP plans were identical to the manual plans. Points below the equality line illustrate a lower dose by the KBP plan for this patient. A) D_mean_ for the hippocampus, B) Volume parameters for the body (measure of the integral dose) D_max_: maximum dose.

The DVH parameters are shown in Table 2 and Figures 1-4.

**Table 2:** Overview of the dosimetric parameters and their comparison The table lists the parameters for the 31 validation patients, planned manually and with three KBP models. Values represent average ± standard deviation across N patients. N<31 indicates organs-at-risk (OAR) was not contoured in all cases, likely because of the large distance to the target. CTV: Clinical target volume; cGy: centigray; D_max_: maximum dose; D_min_: minimum dose; D_mean_: mean dose; V105%: percentage of the volume of the target tissue receiving 105% of the prescribed dose; V95%: percentage of the planning target volume receiving at least 95% of the prescribed dose; cc: cubic centimeters; V50%: volume of the body that receives at least 50% of the prescribed radiation dose; V10%: volume of the body that receives at least 10% of the prescribed radiation dose; V100%: volume of the body that receives at least 100% of the prescribed radiation dose; V30%: volume of the body that receives at least 30% of the prescribed radiation dose; L: left; R: right.

Parameter	N	Manual plan	KBP_V_	KBP_O_	KBP_C_	Significant differences
CTV D_max_ (%)	31	108.6±2.2	108.1±1.9	108.4±2.4	108.3±1.9	
CTV V105% (%)	31	15.2±22.9	3.7±5.9	4.3±6.9	4.5±6.9	Manual vs KBP_V _(p=0.009)/KBP_O_ (p=0.011)/KBP_C_ (p=0.013)
CTV D_min _(%)	31	83.5±12.4	79.7±16.6	81.0±12.3	82.6±13.7	
CTV V95% (worst case)	31	97.1±2.9	97.7±2.2	97.9±2.1	97.7±2.3	Manual vs KBP_O _(p=0.025)/KBP_C_ (p=0.041)
Body V100% (cc)	31	176±150	171±149	176±152	172±150	KBP_V_ vs KBP_O _(p<0.001)
						KBP_O_ vs KBP_C _(p<0.001)
Body V50% (cc)	31	452±299	450±311	457±312	450±310	KBP_V_ vs KBP_O _(p=0.003)
						KBP_O_ vs KBP_C _(p<0.001)
Body V30% (cc)	31	596±345	594±357	600±357	593±355	KBP_V_ vs KBP_O _(p=0.008)
						KBP_O_ vs KBP_C _(p<0.001)
Body V10% (cc)	31	847±409	840±414	843±412	838±413	KBP_O_ vs KBP_C _(p=0.011)
Brainstem D_max _(cGy)	31	3514±2255	3491±2260	3530±2267	3516±2279	
Cochlea_L D_mean _(cGy)	28	997±1448	1009±1583	1001±1573	976±1564	
Cochlea_R D_mean _(cGy)	28	537±1133	458±926	567±1127	556±1128	
Eye_L D_max _(cGy)	29	677±1033	656±1057	672±1079	672±1065	
Eye_R D_max _(cGy)	28	531±950	437±720	511±919	500±889	
Hippocampus_L D_mean _(cGy)	21	790±1077	535±630	584±800	604±888	Manual vs KBP_V _(p=0.040)
Hippocampus_R D_mean _(cGy)	18	729±1234	592±748	646±1015	636±1001	
Lacrimal_L D_mean _(cGy)	16	487±872	526±1029	529±1019	499±1006	
Lacrimal_R D_mean _(cGy)	16	309±642	348±867	327±833	345±843	
Lens_L D_max _(cGy)	28	85±162	88±176	100±198	100±202	
Lens_R D_max _(cGy)	28	90±276	76±277	97±295	91±293	
Optic chiasm D_max _(cGy)	31	2915±2222	2875±2223	2813±2153	2912±2206	KBP_O _vs KBP_C_ (p=0.018)
Optic nerve_L D_max _(cGy)	31	2612±2271	2627±2270	2604±2260	2599±2268	
Optic nerve_R D_max _(cGy)	29	2196±2260	2062±2136	2105±2193	2081±2182	
Pituitary D_max _(cGy)	19	1992±1951	1848±1857	1761±1832	1801±1831	Manual vs KBP_O_ (p=0.033)/KBP_C_ (p=0.020)

The CTV V105% was significantly higher in the manual plans (average±standard deviation of 15.2±22.9% across 31 patients) compared to KBP_V_ (3.7±5.9%), KBP_O_ (4.3±6.9%), and KBP_C_ (4.5±6.9%). KBP_V_ reduced the mean dose to the left hippocampus (Hippocampus_L D_mean_; 535±630 cGy) versus the manual plans (790±1077 cGy). The maximum dose to the pituitary gland (Pituitary D_max_) was reduced in both KBP_O_ (1761±1832 cGy) and KBP_C_ (1801±1831 cGy) compared to the manual plans (1992±1951 cGy).

When comparing the KBP models to each other, KBP_O_ significantly reduced the maximum dose to the optic chiasm (chiasm D_max_; 2813±2153 cGy) compared to KBP_C_ (2912±2206 cGy). No other DVH parameters were significantly different, suggesting small differences resulting from model sizes and different compositions. In two cases the KBP plans exceeded a mean dose (D_mean_) <4500 cGy constraint to the left cochlea that the manual reference plans were able to meet. 

Plan robustness in terms of worst-case CTV V95% (percentage of the planning target volume receiving at least 95% of the prescribed dose) was significantly lower in the manual plans (97.1±2.9%) compared to KBP_O_ (97.9±2.1%) and KBP_C_ (97.7±2.3%). Several measures of the integral dose were not significantly different between the manual and KBP plans, but there were some slight differences between KBP models (see Table 2 for details). 

In the blind review by the physician, all the 31 KBP_C_ plans (as well as all the manual reference plans) were considered clinically acceptable. When stating a preference, the physician favored the KBP_C_ plans in 12 out of 31 cases. A tie was scored for 13 out of the 31 plans, while the manual plans were preferred in six out of 31 cases. 

## Discussion

Several KBP models for targets in the brain and base-of-skull treated with IMPT were developed with different training sets and model sizes. To our knowledge, this is the first reported KBP model for brain and base-of-skull IMPT used to create clinical plans and investigate the effects of KBP model size and composition. 

Published literature generally recommends developing KBP models with a large database of plans, and separating the training and validation sets [11,23-25]. In this study, we investigated the consequences of having only a small dataset and using identical plans for training and validation. This scenario can become necessary when plan availability is limited. 

Compared to the previously-treated manual reference plans, some minor improvements were observed in all KBP plans such as lower V105% and slight reductions in OAR doses, demonstrating the potential of KBP. We found that the small-dataset KBP model (KBP_V_) produced plans that were comparable to those generated by both KBP_O_ and KBP_C_, where KBP_O_ had a training set completely independent of the validation set, and KBP_C_ had the largest model size. 

This study included a blind physician review which confirmed that all KBP_C_ plans were clinically acceptable, and in the majority of cases (25/31) KBP_C_ plans were considered to be of higher quality or similar to manually-generated plans. These findings demonstrate the possibility of improving the quality of IMPT treatment planning with KBP, even when only a limited number of treatment plans are available for training the models. 

This study has several limitations. The blind review was only done for the manual plans in comparison to the KBP_C_ model (the final model currently in clinical use). Given the small differences in DVH parameters between the models, a blind four-way review was not deemed feasible, as the reviewer likely would have been able to identify the manual plan out of the four. 

Another limitation in the model is shown in the results where the KBP plans for two patients exceeded a clinical constraint for the cochlea where the manual plan showed an acceptable dose. This underscores the need for a thorough review of the plan before treatment to evaluate whether additional organ sparing may be achievable. Furthermore, planners should evaluate whether localized optimization structures can help with local hot and cold spots. In this study, a broad additional upper body objective was added in a continue optimization step which reduced hot spots, but additional improvements may be possible with human refinement. 

The low impact of model size on the resulting KBP plan quality found here aligns with some findings in the previously published literature [26,27], while other studies see plan improvement with larger models [28]. One hypothesis for our finding is the labor-intensive development of these models. Many fixed objectives and priorities were created and tuned by the developers to ensure clinical constraints were achieved (an example would be the 4100 cGy upper objective on the spinal cord, see Table 1). The KBP model was allowed to generate additional objectives, albeit typically at a lower priority than the fixed and tuned objectives. With careful tuning, this approach enables higher quality plans but reduces the impact of the training set. If the KBP model relied solely or mostly on objectives generated by the KBP routine, as seen in some studies [14,18,29,30], the composition of the KBP training set may have had a more substantial impact on the resulting plan quality than observed here. An additional note is that the KBP solution used in this study requires a minimum of 20 cases for each structure before a DVH estimation can be generated. We ensured that this minimum requirement was met for even the smallest model. 

Therefore, the authors would like to caution clinics considering KBP implementation to perform continuous quality control and discuss strategies for implementing and evolving KBP models over time [13,28,29]. It cannot be ruled out that larger differences may emerge with varying model sizes or while mixing validation and training cases, particularly when applied to other disease sites or to different sets of optimization objectives. 

## Conclusions

In this study, we developed and compared several KBP models for targets in the brain and base-of-skull treated with IMPT. KBP models generated plans with minimal dosimetric differences to manual planning. The high acceptance rate of KBP plans in the blind review further confirmed the high performance of these models and the clinical viability of the KBP plans. Minimal differences between plans from the three KBP models suggest that clinics can train and validate KBP models using the same plans when only limited data is available. These findings support the implementation of KBP in IMPT where often only a few plans are available. 
